# Is peer support a tipping point for the opioid use disorder crisis in Appalachia? Research holds the answer

**DOI:** 10.1186/s12954-024-01041-7

**Published:** 2024-06-25

**Authors:** Kimberly Horn, Stephanie M. Mathis, Lara Nagle, Angela Hagaman, Mary Beth Dunkenberger, Robert Pack

**Affiliations:** 1 Virginia Tech Institute for Policy and Governance, 201 W. Roanoke Street, Blacksburg, VA 24061 USA; 2 East Tennessee State University Addiction Science Center, 2109 West Market Street, Johnson City, TN 37604 USA

**Keywords:** Opioid use disorder (OUD), Peer recovery support services (PRSS), Central Appalachia, Community-engaged research

## Abstract

**Background:**

The present commentary highlights the pressing need for systematic research to assess the implementation and effectiveness of medications for opioid use disorder, used in conjunction with peer recovery support services, to improve treatment outcomes for individuals with opioid use disorder in Central Appalachia. This region, encompassing West Virginia, Eastern Kentucky, Southwest Virginia, East Tennessee, and Western North Carolina, has long grappled with a disproportionate burden of the opioid crisis. Due to a complex interplay of cultural, socioeconomic, medical, and geographic factors, individuals in Central Appalachia face challenges in maintaining treatment and recovery efforts, leading to lower success rates.

**Approach:**

To address the issue, we apply an exploratory approach, looking at the intersection of unique regional factors with the utilization of medications for opioid use disorder, in conjunction with peer recovery support services. This combined treatment strategy shows promise in addressing crucial needs in opioid use disorder treatment and enhancing the recovery journey. However, there are significant evidence gaps that need to be addressed to validate the expected value of incorporating peer support into this treatment strategy.

**Conclusion:**

We identify nine obstacles and offer recommendations to address the gaps and advance peer recovery support services research. These recommendations include the establishment of specific partnerships and infrastructure for community-engaged, peer recovery support research; improved allocation of funding and resources to implement evidence-based practices such as peer support and medication-assisted treatment; developing a more precise definition of peer roles and their integration across the treatment and recovery spectrum; and proactive efforts to combat stigma through outreach and education.

## Introduction

Opioid use disorder (OUD) and the resulting surge in overdose fatalities have reached a critical level as a nationwide public health crisis, with Central Appalachia emerging as a particularly affected region. Central Appalachia encompasses Southeastern Ohio, West Virginia, Eastern Kentucky, Southwest Virginia, East Tennessee, and Western North Carolina. From 2019 to 2020, there was a nearly 30% increase in drug overdose deaths nationally, representing an exponential increase since 1999 [1]. Tragically, opioids have been implicated in the premature deaths of 564,000 individuals between 1999 and 2020 [2]. Overdose and overdose deaths further increased from 2020 to 2022, exacerbated by the COVID-19 pandemic and the resulting impacts of reduced access to harm reduction and treatment services, especially in rural areas such as Central Appalachia [3]. These opioid overdose deaths are part of a more extensive drug overdose epidemic that has persisted for over four decades, with a steep escalation in fatalities over time [4].

While the opioid crisis is a national public health emergency, Appalachia–particularly the Central Appalachian region–has borne a disproportionate burden for many years [5–8].

Between 2008 and 2014, Central Appalachia experienced drug poisoning mortality rates 154% higher than the national average and 79% higher than the average within the Appalachian region [6, 7]. In 2021, Appalachia experienced significantly higher overdose-related mortality rates for people aged 25–54, with rates 72% greater than the rest of the country [8]. These rates reached 45.6 and 20.4 per 100,000 people, primarily driven by opioid-related deaths, which accounted for over 65% of all fatalities in the region [8].

The most recent data available through September 2023 from the Centers for Disease Control (CDC) National Center for Health Statistics indicate that drug overdose deaths continue to increase, with opioids accounting for the largest percentage of these deaths [9]. The CDC characterizes three waves of opioid overdose deaths, beginning in the 1990’s with the rise of prescription opioids, again in 2010 due to heroin use, and again in 2013 with the introduction of synthetic opioids [10]. The growing prevalence of fentanyl over the past few years has radically altered the landscape of substance use disorders because of its potency and addition to counterfeit drugs as well other drug supplies, suggesting that a fourth wave may be occuring [11]. This highlights an urgent need to enhance our understanding of effective treatment approaches.

Individuals’ access to and engagement in treatment has improved in Appalachia; still, it is estimated that fewer than 20% engage in treatment for opioid use disorder (OUD) [12]. MOUD includes medications such as methadone (a full opioid agonist), buprenorphine (a partial opioid agonist available in different formulations like Suboxone®), and naltrexone (an opioid antagonist). MOUD, especially but not exclusively combined with counseling, is considered the gold standard of care for treating OUD [13, 14]. It helps individuals recover by stabilizing their opioid cravings, reducing the risk of overdose, and supporting them in achieving and maintaining abstinence from illicit opioids. MOUD programs combine medication(s) with counseling, therapy, and other supportive services to provide comprehensive care for individuals with OUD [15–18].

Increasingly, support services are delivered by people with lived experience with substance use disorder (SUD) who are now in sustained recovery. Such peer recovery support services (PRSS) are essential for improving recovery outcomes and are employed at the intersection of community-based harm reduction and treatment [19]. Peer support or peer-supported services encompass a range of mental health and substance use support options, including inpatient, outpatient, digital, and community-based services. Peer recovery support professionals are trained to use their lived experience with mental illness and SUD to help others with these challenges to navigate the network of resources that may be available, in addition to providing emotional support, advocacy, coaching, and mentorship.

In the United States, over 30,000 peer support specialists, known by various titles such as peer providers, peer coaches, peer recovery specialists (PRS), or peer mentors, offer services that can be reimbursed through Medicaid in 43 states [20]. Expanding peer support has led to significant advancements in both its availability and diversity [20]. Within the study area, states such as Virginia offer Medicaid reimbursements for PRSS under specific conditions, aligning with state certification requirements [21]. These requirements, which vary by state, set standards for PRSS and ensure a level of quality and accountability in OUD treatment.

Although these services are highly regarded for their contribution to overall recovery among individuals with OUD, the adoption, and use of PRSS as a strategy is not consistent across Appalachian organizations and community members, including those with OUD, in part due to the limited published evidence on outcomes, content, and practices of PRSS, particularly within the context of MOUD. Furthermore, PRSS need more consistent funding and integration by treatment providers and insurers in Central Appalachia. Notably, with the introduction of the National Institutes of Health (NIH) Helping to End Addiction Long-term Initiative (HEAL) [22], new and emerging research suggests that PRSS could play a pivotal role in addressing the OUD crisis [23, 24]. Because a robust evidence base is lacking, research is essential to understand and prove the impact of PRSS.

Given the pressing need for research on peer support in combination with MOUD, and more specifically, the additive effect of PRSS in Central Appalachia, it is imperative to establish a comprehensive research plan that strengthens the region's critical research-to-practice infrastructure. This entails enhancing researcher capacity, involving clinical sites and PRSS providers, and providing data support to conduct reliable and valid controlled studies on PRSS. The plan must also address socio-cultural factors that impede the adoption of PRSS across regional service networks, such as divergent community perspectives on abstinence-only approaches, the stigma surrounding MOUD, particularly as a harm reduction strategy, and the limited knowledge of evidence-based treatment [25]. This commentary emphasizes two streams of inquiry: investigations assessing the combined effectiveness of PRSS with MOUD on recovery outcomes, and studies examining the operationalization and implementation of these services within clinical settings. Strategically funded and conducted effectiveness and implementation research can inform the outcomes and expansion of recovery support services, particularly PRSS, and advance the recovery journey among individuals in Central Appalachia receiving MOUD treatment.

## Discussion

We separate our discussion into two focal areas: the current evidence base substantiating the effectiveness of PRSS in advancing recovery milestones, and the emerging recommendations for comprehensive research to solidify PRSS as an evidence-based practice.

## The evidence base 

According to the Substance Abuse and Mental Health Services Administration (SAMHSA), recovery is a dynamic process characterized by positive health and overall well-being changes, enabling individuals to lead self-directed lives and strive for their maximum potential [19]. Most recently, Ashford and colleagues defined recovery as “an individualized, intentional, dynamic, and relational process involving sustained efforts to improve wellness” [26]. A comprehensive study conducted by Kelly and colleagues in 2017 found that 9.1% of adults in the United States, representing tens of millions of Americans, had resolved a significant alcohol or substance use problem [27]. Among this population, more than half (53.9%) reported utilizing treatment or mutual aid to support their recovery [27].

Research on recovery attempts indicates that individuals typically make a median of two, and a mean of five, attempts before achieving sustained recovery [28], often requiring long-term recovery and peer support services [29, 30]. The latter are provided by both compensated and volunteer roles, such as patient navigators, certified peer recovery specialists (CPRS), recovery community members and sponsors, and recovery housing residents and house leaders, among others [19, 30]. The care paradigm for individuals with OUD is shifting from a focus on pathology and intervention to a long-term recovery approach [31]. In conjunction with efforts to expand access to MOUD, various agencies, private and public health insurance providers, hospital systems, and others are promoting diverse forms of PRSS.

During the past several years, PRSS staffing has been primarily supported by programs like SAMHSA’s State Targeted Response to the Opioid Crisis or subsidized by discretionary funds or other revenue-producing service areas, as Medicaid and other insurance providers rarely cover the actual cost of PRSS. Some states, such as Virginia in 2022, passed legislation increasing the reimbursement rates for peer recovery and family support services in private and public community-based recovery services settings from $6.50 to $19.50 per 15 min for individuals and from $2.70 to $8.10 per 15 min for groups [32]. The legislative explanation for this change notes that “[c]urrent rates are so low that few, if any, community services boards can afford to bill for Medicaid reimbursement for these services. Research demonstrates that these services provide successful interventions for individuals in crises and in overcoming addiction” [32]. Some states are also using opioid abatement settlement dollars to support PRSS.

While emerging research suggests that recovery support services positively impact individuals, families, and communities [33], there is a significant gap in conducting and disseminating research to inform the content, practice, and long-term efficacy of these services. Standards for MOUD are well-defined, yet there is much to learn regarding the type, frequency, and duration of supplemental recovery support services [34]. Furthermore, there needs to be more evidence on how these support services function, how they should be organized, delivered, and sequenced, their potential return on investment, and models for sustaining their effectiveness. In alignment with preliminary evidence on PRSS roles in Central Appalachia [35], we posit that combining MOUD with peer support should be remarkably beneficial for individuals with OUD residing in Central Appalachia. There is exciting work ahead to empirically validate the impact of PRSS in OUD treatment. Establishing a strong evidence base is essential for the integration of PRSS as a recognized component of comprehensive OUD treatment strategies.

## A closer look at medications for opioid use disorder in central *Appalachia*

Retention in MOUD therapy has demonstrated a reduction in opioid-related mortality by up to 50% [36–38]. The SAMHSA State Targeted Response to the Opioid Crisis program has played a critical role in expanding MOUD access [39], particularly in high-need areas [40–42]. MOUD clinics are typically part of opioid treatment programs (OTPs) or office-based opioid treatment (OBOT) services. OTPs provide outpatient therapy and dispense methadone on-site while adhering to SAMHSA regulations. Counseling is an essential component of OTPs, varying in intensity based on treatment adherence and program duration. Conversely, outpatient treatment with buprenorphine-containing products can be delivered in various settings, from primary care facilities to OBOT clinics specializing in OUD care, where over 50% of patients seek treatment. Physicians are eligible to prescribe buprenorphine products for MOUD in all states, while nurse practitioners and physician assistants can prescribe it in most. Patients then fill their prescriptions at a pharmacy. Many OBOTs offer on-site counseling or collaborate with counseling facilities.

Though sparse, existing evidence suggests that despite the established efficacy of MOUD, patient retention in Appalachian regions remains a significant challenge [43]. Low utilization of MOUD in Appalachian populations can be attributed to factors such as limited treatment access, lack of awareness, cultural influences, economic challenges, healthcare workforce shortages—especially among providers with adequate training in evidence-based treatments—regulatory barriers, transportation difficulties, limited insurance coverage, and social isolation. Stigma associated with OUD treatment presents a pervasive obstacle to both access and retention in treatment programs. Stigmatization can dissuade individuals from seeking help and undermine public support for funding and policy initiatives. It is essential to address stigma through education and community engagement to create an environment where treatment is seen as a legitimate and necessary medical intervention.

Furthermore, limited educational and economic opportunities contribute to systemic poverty, significantly influencing higher OUD rates and overdose incidents [44]. These barriers hinder individuals from seeking and accessing MOUD treatment, emphasizing the need for targeted interventions, awareness campaigns, healthcare access improvements, and culturally sensitive approaches to address this issue effectively in these regions.

## Unique relevance of peer recovery support services (PRSS) in central *Appalachia*

PRSS can refer to the process of providing and receiving non-clinical assistance to achieve long-term recovery from SUDs [45]. Peers, individuals in recovery from OUD or co-occurring mental health disorders, possess unique qualifications based on their lived experience and experiential knowledge [30, 31, 45]. Grounded in theory and mutual support traditions, peer support relies on mutual identification and encompasses educational, coaching, and various forms of support, such as affiliational, emotional, informational, and instrumental support [29, 46]. PRSS, with its person-centered and strengths-based approach, can contribute to developing recovery capital [29, 47, 48]. Despite limited evidence, PRSS implementation is growing in clinical and community settings [30, 45]. Since 2004, peer support in addiction treatment has evolved significantly. This evolution includes the emergence of specialized roles, standardized training and certification processes, reimbursement mechanisms, competency frameworks, and fidelity assessments. Peer support has expanded internationally and is recognized as an essential component of addiction treatment services [20]. Central Appalachia has benefitted from a significant increase in trained PRSS individuals; however, there needs to be more understanding of this valuable group, including their practice settings and approaches that serve the region [35]. Advancing PRSS research is crucial given the expansion of PRSS in various settings and their unique potential to address gaps in care for individuals receiving MOUD treatment.

Central Appalachia faces distinct challenges in OUD treatment compared to other low socioeconomic status regions in the United States [44]. These challenges of rurality are exacerbated by terrain and travel distances and lack of trust in traditional health professionals. PRSS can help mitigate these barriers by virtue of their local presence, cultural competence, and position as role models, mentors, advocates, and motivators [49]. Peers can engage individuals beyond traditional boundaries [30] and extend the reach of care beyond clinical settings [29]. Furthermore, PRSS can be delivered across various settings and by different service providers throughout the recovery continuum [30, 35, 45, 46]. Additionally, PRSS may offer a cost-effective alternative to services provided by licensed medical or counseling professionals [31].

Unfortunately, the scientific and practical knowledge about PRSS in the context of MOUD clinics such as OBOTs/OTPs is limited by significant research gaps, particularly in terms of experimental trials and stages of treatment [50]. The methodology behind PRSS research is distinct due to its emphasis on qualitative insights and lived experiences as key data points. These methods, which may include narrative analysis and participatory research designs, prioritize the subjective aspects of recovery and are integral to understanding the full impact of PRSS. Qualitative findings suggest that PRSS can enhance relationships with providers and social support networks, reduce SUD and relapse rates, and increase treatment satisfaction and retention. Additionally, other emergent research suggests that it *is* possible to identify and conceptualize the vast array of PRSS, a critical step for adequately measuring PRSS outcomes across numerous service settings in Central Appalachia [35].

Longitudinal evidence in this area is scarce [30, 45] and can be a goal. But in order to achieve this goal, methodological and measurement concerns that hinder definitive conclusions must be addressed [35, 44]. Challenges with separating the impacts of PRSS from other forms of support, use of various definitions and measured outcomes related to PRSS, inclusion of various populations, and absence of suitable comparison groups, among others, contribute to these limitations [30, 45, 46]. Despite the expanded access to MOUD and PRSS in many regions, research specifically focused on PRSS in coordination with MOUD remains notably lacking in Central Appalachia.

## Recommendations

To expedite the urgently needed research on PRSS in Central Appalachia, we present recommendations to overcome the current obstacles and accelerate progress in peer recovery support research. Among the most pressing obstacles are regional stigma around MOUD and systemic issues in service integration of PRS. While these challenges are different from evidence gaps regarding intervention effectiveness, they too can be addressed through targeted research initiatives.

While this may not be an exhaustive list, the proposed strategies encompass several key areas, including research capacity, community engagement, data harmonization, funding, integration of PRSS into the treatment continuum, evidence-based practices (EBPs), stigma reduction, and collaboration among stakeholders. Each recommendation offers unique perspectives and action items for building research capacity to tackle the OUD crisis in the region.

## Build multi-stakeholder capacity to conduct community-engaged PRSS research, including universities, health systems, community and state partners, and PRSS providers

### Impediments to progress

Opinions, stigma, and lack of education regarding OUD treatment and harm reduction options can act as barriers to conducting rigorous and impactful PRSS research in Central Appalachia. Professionals and community members may also have limited knowledge and expertise in PRSS research.

### Action items to facilitate progress

It is crucial to enhance research capacity by providing education and training opportunities. This includes engaging universities, health systems, community and state partners, and PRSS providers to foster collaboration and knowledge exchange. Offering education and training programs on topics such as harm reduction and community-engaged research can empower early career and student investigators as well as community-based peer specialists. Establishing partnerships with colleges and universities in the region will also be valuable in developing tailored programs and resources.

### Example

Implement recovery research certificate programs specifically designed for Central Appalachia that offer open access and credit and non-credit bearing options. Recovery research certificate programs could be expanded to incorporate harm reduction principles, ensuring a comprehensive understanding of the diverse approaches to SUD treatment. Alternatively, if the aim is to maintain a focused curriculum, these programs should clearly define 'recovery supports' to align with the specific competencies required for PRSS roles. Such offerings are critical to educate professionals and community members on PRSS research methods and harm reduction strategies.

## Enroll research cohorts of MOUD clinics and PRSS professionals to foster commitment to and engagement in rapid completion of PRSS studies

### Impediments to progress

Limited awareness about the importance of quasi-experimental and controlled clinical trials engaging MOUD clinics and PRSS professionals hinders their active participation and engagement in research activities.

### Action items to facilitate progress

Creating a network of dedicated sites for PRSS trials in Central Appalachia can help address this issue. This network will facilitate communication and collaboration among addiction treatment providers, including those operating OTPs and OBOTs. By involving these clinics and professionals, valuable insights into the thoughts, opinions, and experiences of addiction treatment providers in Central Appalachia can be obtained, leading to improved ways of serving individuals, families, and communities affected by SUD.

### Example

Establish a network of MOUD clinics and PRSS professionals in Central Appalachia dedicated to participating in PRSS trials and research activities. This network can serve as a platform for sharing best practices, promoting and exchanging knowledge, and fostering a commitment to research.

Establishing network sites for trials presents logistical and collaborative challenges, particularly in regions with diverse healthcare landscapes like Central Appalachia. These challenges include aligning protocols across sites, ensuring data consistency, and engaging stakeholders with varying levels of resources and expertise. To mitigate these issues, we propose integrating trial network development with initiatives like the NIH HEAL program, which already provides infrastructure and funding designed to address the opioid crisis. Leveraging HEAL resources and frameworks could streamline the creation of network sites, promote uniform standards, and foster a cooperative environment conducive to high-quality, multi-site research.

## Establish processes and technologies to harmonize data and research efficiencies that advance PRSS studies across a range of partners

### Impediments to progress

Lack of data harmonization and limited resources for data analysis hinder the sharing, use, and dissemination of research data. This leads to inefficiencies and a lack of collaboration among research partners.

### Action items to facilitate progress

Implement processes and technologies that facilitate data harmonization and improve research efficiencies. This includes establishing a data sharing platform, identifying common PRSS data elements, developing Data Use/Trust Agreement templates, and implementing a unified human subjects research review process. By enhancing data sharing and analysis capabilities, research productivity and dissemination of new information can be increased. It is also important to provide support systems, including infrastructure, skilled human capital, and clear plans for preparing joint trial protocols and scholarly products, to overcome the barriers associated with limited data analytic resources.

### Example

Create an approachable “Data Lake” that offers low-cost, scalable, and secure Health Insurance Portability and Accountability Act (HIPAA)-compliant storage. This infrastructure should support search and analysis capabilities for various data types, enabling researchers to collaborate and access data efficiently. The proposed Data Lake is a secure, centralized repository that adheres to the highest standards of patient privacy and data security. To differentiate from other data harmonization efforts, it will specifically allow for the aggregation of data across multiple sites without compromising individual privacy. This platform will enable the comparative analysis of PRSS effectiveness across diverse settings while safeguarding sensitive patient information.

## Identify priority research questions through researcher-community engagement

### Impediments to progress

Lack of alignment between research questions and community needs hampers the relevance and impact of PRSS studies in Central Appalachia.

### Action items to facilitate progress

Foster proactive engagement between researchers and the community driven by peer workers' perspectives to identify priority research questions. Community members refers to a broad spectrum of stakeholders including individuals in recovery, family members affected by OUD, healthcare providers, and local policymakers. By involving the community in the research process, including individuals with lived experiences of OUD and community organizations that serve those individuals, researchers can gain valuable insights into the specific challenges and needs of the local population. This engagement will ensure that research efforts are targeted towards addressing the most pressing issues faced by the community.

### Example

Conduct focus groups, community forums, and surveys to gather input from individuals with OUD, community leaders, and other stakeholders to identify research questions that align with their needs and priorities. To generate ideas beyond focus groups, we propose the use of online platforms for virtual town halls, enabling wider and more inclusive participation. These multi-stakeholder efforts also aid in helping people hear and grasp different viewpoints, related to Recommendation #9.

## Conduct rapid-cycle intervention research using rigorous designs

### Impediments to progress

Traditional research designs with lengthy timelines impede the timely implementation and evaluation of PRSS with MOUD interventions.

### Action items to facilitate progress

Adopt rapid-cycle intervention research approaches that allow for quick implementation and evaluation of PRSS interventions. These designs emphasize iterative and adaptive processes, enabling researchers to make modifications based on real-time feedback and results. By employing rigorous designs in tandem, such as randomized controlled trials (RCTs), the effectiveness and implementation of PRSS interventions can be assessed more efficiently. This recommendation advocates for an innovative blend of research designs, encompassing both rapid-cycle designs for immediate, actionable feedback, and RCTs for robust, long-term efficacy data. This dual approach allows for tailored interventions responsive to the distinct needs of rural and urban settings within Central Appalachia, catering to the diverse demographics and cultural nuances of each sub-population.

### Example

Develop adaptive trial design guidelines for researchers that provide recommendations for tailoring PRSS interventions to different settings and populations. This will ensure that interventions are flexible and can be adjusted based on the unique needs and characteristics of Central Appalachia.

## Increase funding and resources

### Impediments to progress

Adequate funding is essential to support the development, implementation, and sustainability of PRSS programs. Federal, state, and local governments should prioritize funding for PRSS initiatives in Appalachia, considering the high burden of the OUD crisis in the region. Additionally, limited resources hinder the training and continuing education of peer support specialists and the establishment of necessary infrastructure for effective PRSS.

### Action items to facilitate progress

Increase funding allocations from federal, state, and local sources specifically earmarked for PRSS initiatives in Central Appalachia. This includes supporting grant programs, creating dedicated funding streams, and leveraging public–private partnerships, including advocating for private insurance coverage. Additionally, allocate resources for the training and education of peer support specialists and invest in the infrastructure needed to deliver high-quality PRSS.

Enhanced funding through Medicaid reimbursements should also bolster research by enabling more comprehensive data collection and analysis. This financial support will facilitate in-depth, longitudinal studies to establish the long-term efficacy of PRSS and inform policy and funding decisions.

### Example

Establish a grant program (e.g., state block grants) through a collaboration between federal agencies, state governments, and private foundations to provide funding for PRSS initiatives in Central Appalachia. This program should prioritize initiatives that demonstrate a strong evidence base, community engagement, and the potential for long-term sustainability.

## Integrate PRSS into the treatment continuum

### Impediments to progress

Although PRSS have grown significantly over the past decade, they are not fully integrated into the broader treatment continuum for SUD in Central Appalachia. Limited collaboration and coordination between treatment providers and organizations that provide PRSS impede seamless referrals, warm handoffs, and continuity of care.

### Action items to facilitate progress

Promote collaboration and partnerships between treatment providers, health systems, and PRSS organizations to integrate PRSS into the treatment continuum. Develop protocols and processes for seamless referrals, warm handoffs, and ongoing coordination of care. This integration will ensure that individuals receiving MOUD or other forms of SUD treatment can access comprehensive support that includes PRSS. By establishing clear protocols for referral and follow-up between PRSS providers and traditional healthcare services, we can create a seamless continuum of care that maximizes both resources and patient outcomes. Related factors include increasing insurance reimbursement rates to bill for PRSS at sustainable levels, as well as clear standards and definitions of the roles and responsibilities for PRSS in a variety of care settings along the continuum.

### Example

Establish collaborative agreements between addiction treatment clinics, hospitals, and PRSS organizations in Central Appalachia to facilitate seamless referrals and warm handoffs. Develop standardized protocols and shared electronic health record systems to enhance communication and coordination of care between treatment providers and PRSS providers.

## Evaluate and disseminate evidence-based practices

### Impediments to progress

Although PRSS have shown promise in supporting OUD recovery, there is a need for further research to establish EBPs and guidelines. The lack of rigorous evaluation hampers decision-making regarding the implementation and scaling of PRSS programs.

### Action items to facilitate progress

Foster collaboration between researchers and practitioners to conduct rigorous evaluations of PRSS. This includes assessing their impact on treatment retention, abstinence rates, social support, and overall well-being and recovery capital. Evidence-based practices need to be harmonized with existing research initiatives to avoid duplication. By consolidating efforts, we can create a more focused and potent strategy for evaluating PRSS interventions and disseminating best practices. With access to an evidence base, stakeholders can make informed decisions about the design, implementation, and scaling of PRSS programs in Central Appalachia.

### Example

Establish a research consortium comprised of researchers, treatment providers, PRSS organizations, and community members in Central Appalachia. This consortium will collaborate on conducting rigorous evaluations of PRSS interventions and disseminate the findings to inform practice and policy.

## Address stigma and promote education

### Impediments to progress

The stigma surrounding OUD and MOUD remains a significant barrier to the utilization of PRSS and other supportive services. Limited education and awareness about OUD, MOUD, and the value of peer support contribute to the perpetuation of stigma.

### Action items to facilitate progress

Develop targeted education campaigns to combat stigma and promote accurate information about OUD, MOUD, and the benefits of peer support. These campaigns should target healthcare providers, policymakers, community leaders, and the general public. By increasing awareness and understanding, stigma can be reduced, and individuals seeking recovery can access the support they need. Stigma reduction campaigns should align with existing initiatives by incorporating evidence-based strategies that have proven successful in other contexts. This targeted approach will address specific misconceptions about PRSS and MOUD, leveraging lessons learned from past efforts to maximize impact.

### Example

Collaborate with local organizations, healthcare providers, and community leaders to develop and implement evidence-based stigma reduction campaigns tailored to Central Appalachia. These campaigns can utilize various mediums, such as social media, public events, and community workshops to disseminate accurate information, challenge misconceptions, and promote the value of PRSS.

For each of our recommendations, it is essential to consider the distinct socio-economic and cultural backdrop of Central Appalachia—a region with unique challenges such as geographical isolation, prevalent substance use, and a historically-strained relationship with healthcare systems. Collaboration in this context shapes the implementation and effectiveness of PRSS and must inform how we tailor our approaches to meet community needs. Therefore, our recommendations are not just generic suggestions but are deeply rooted in the lived experiences of Central Appalachians, reflecting a conscientious effort to address specific barriers and leverage stakeholders’ inherent strengths. By aligning our strategies with the region's unique characteristics, we aim to ensure that our proposed efforts are culturally sensitive, practically applicable, and likely to be embraced by the individuals they are intended to support.

## Conclusion

The OUD crisis in Central Appalachia continues to have devastating consequences, marked by disproportionately high rates of overdose deaths and limited access to effective treatment. The utilization of PRSS shows promise in enhancing the recovery journey and addressing critical gaps in care for individuals with OUD. However, the pressing need for comprehensive research cannot be overstated, as it is imperative to document effectiveness and unravel the barriers and challenges entailed in accessing and implementing PRSS in Central Appalachia, especially within the context of MOUD treatment.

Through collaborative discussions among researchers, practitioners, and individuals in recovery, several key recommendations have emerged to expedite meaningful research in this area. These recommendations span various domains, encompassing research capacity, community engagement, data harmonization, funding, integration of PRSS into the treatment continuum, EBPs, stigma reduction, and collaboration among stakeholders. By putting these recommendations into action, we contend that the existing obstacles can be surmounted, ushering in accelerated progress to confront the OUD crisis in Central Appalachia.

To comprehensively evaluate the efficacy of PRSS, research must include qualitative studies that include the lived experiences of peer providers. This will provide nuanced insights into the sociocultural dynamics influencing their work. Multi-level and multivariate models should also be employed to analyze these experiences in the context of the broader healthcare ecosystem and the specific cultural milieu of Central Appalachia.

With a concerted effort to strengthen the research infrastructure, engage a spectrum of stakeholders, and address the unique sociocultural factors influencing PRSS adoption, there is a real opportunity to enhance access to effective treatment and support services for individuals grappling with OUD in the region. By bridging the gap between research and practice, comprehensive research endeavors can pave the way for evidence-based strategies, improved recovery outcomes, and a meaningful contribution to public health initiatives combating the opioid crisis in Central Appalachia.

## Data Availability

Not applicable.

## Abbreviations

EBP: Evidence-Based Practice
HIPAA: Health Insurance Portability and Accountability Act
HEAL: Helping to End Addiction Long-term Initiative
MOUD: Medications for Opioid Use Disorder
NIH: National Institutes of Health
OBOT: Office-Based Opioid Treatment
OTP: Opioid Treatment Program
OUD: Opioid Use Disorder
PRS: Peer Recovery Specialist
PRSS: Peer Recovery Support Services
RCT: Randomized Controlled Trial
SAMHSA: Substance Abuse and Mental Health Services Administration
SUD: Substance Use Disorder
